# Experimental Infection of Bundibugyo Virus in Domestic Swine Leads to Viral Shedding with Evidence of Intraspecies Transmission

**DOI:** 10.1155/2024/5350769

**Published:** 2024-01-09

**Authors:** Charles E. Lewis, Mathieu M. Pinette, Steven M. Lakin, Greg Smith, Mathew Fisher, Estella Moffat, Carissa Embury-Hyatt, Brad S. Pickering

**Affiliations:** ^1^Department of Veterinary Microbiology and Preventive Medicine, College of Veterinary Medicine, Iowa State University, Ames, Iowa, USA; ^2^Interdepartmental Microbiology Program, College of Agriculture and Life Sciences, Iowa State University, Ames, Iowa, USA; ^3^National Centre for Foreign Animal Diseases, Canadian Food Inspection Agency, Winnipeg, Manitoba, Canada; ^4^Scientific Liaison Services Section, Foreign Animal Disease Diagnostic Laboratory, National Veterinary Services Laboratories, Animal Plant Health Inspection Service, United States Department of Agriculture, Orient Point, New York, USA; ^5^Department of Medical Microbiology and Infectious Diseases, University of Manitoba, Winnipeg, Manitoba, Canada

## Abstract

The *Ebolavirus* genus contains several of the deadliest zoonotic viruses known. One of these, Bundibugyo virus (BDBV), has been the causative agent of two outbreaks of human disease that have resulted in 211 known cases with a case fatality rate of 33.6%. Although bats are routinely implicated as the possible reservoir species for the ebolaviruses, the source of infection for index cases in almost all outbreaks is unknown with only limited epidemiological evidence directly linking human cases to bats. This lack of evidence leaves open the possibility that maintenance of one or more of these viruses could involve multiple host species or more complex spillover dynamics. Domestic pigs have been found naturally infected with Reston virus (RESTV) and are experimentally susceptible to infection with Ebola virus (EBOV), two other members of the *Ebolavirus* genus. Infection of pigs resulted in shedding of infectious virus with subsequent transmission to naïve animals being documented, including transmission to humans for RESTV and to nonhuman primates for EBOV. The susceptibility and subsequent viral shedding and pathogenesis of domestic pigs to other ebolaviruses and the potential role this species may play in virus ecology, spillover dynamics, and human public health risk is unknown. For these reasons, we conducted a series of studies aimed at determining the susceptibility of domestic pigs to BDBV thereby demonstrating that pigs are not only susceptible to experimental infection but that the development of productive infection, tissue dissemination, and shedding of infectious virus can also occur while animals remain clinically normal. The role of pigs as a possible interim or amplifying host for ebolaviruses is a concern for both human public health and food security.

## 1. Introduction

The *Ebolavirus* genus, family *Filoviridae*, is currently composed of six enveloped, filamentous, negative sense RNA viruses [1]. Of these, four are known to be zoonotic pathogens capable of causing severe, potentially lethal, disease in humans: Ebola virus (EBOV, *Zaire ebolavirus*), Sudan virus (SUDV, *Sudan ebolavirus*), Bundibugyo virus (BDBV, *Bundibugyo ebolavirus*), and Taï Forest virus (TAFV, *Tai Forest ebolavirus*). In contrast, Reston virus (RESTV, *Reston ebolavirus*) is considered as avirulent, yet capable of infecting humans [2]. The most recent addition to the genus, Bombali virus (BOMV, *Bombali ebolavirus*), has unknown zoonotic potential as of 2018 [1, 3].

BDBV was discovered in 2007 as the causative agent in an outbreak of lethal hemorrhagic disease originating in Kabango village in the Bundibugyo district of western Uganda [4, 5]. Since its discovery, BDBV has reemerged once causing the 2012 outbreak in the Isiro Health District of the Democratic Republic of Congo [6]. Combined, there have been a total of 211 human cases with 71 deaths, demonstrating a case fatality rate of 33.6% [2]. Although bats are routinely implicated as the possible reservoir for the ebolaviruses, the source of infection for index cases in almost all outbreaks is unknown with only limited epidemiological evidence directly linking humans and bats [7]. This lack of evidence allows for the possibility that ebolavirus maintenance could involve multiple host species or more complex spillover dynamics [7, 8].

In 2008, the discovery of domestic pigs naturally infected with RESTV in the Philippines expanded our understanding of the potential ebolavirus host range [9]. During this epizootic, transmission of virus from pigs to humans was documented, albeit without the development of clinically apparent disease in the people involved [9, 10]. Subsequent to this discovery, experimental infections of pigs with EBOV, a human pathogenic virus, demonstrated the susceptibility of pigs to the African-origin ebolaviruses [11]. EBOV replicated to high titers in these animals and showed a predilection for the respiratory tissues resulting in severe pulmonary pathology with shedding of infectious virus by the oral and nasal routes. In addition, EBOV was shown to be transmissible between pigs [11, 12]. Further studies demonstrated that experimentally infected pigs were also capable of transmitting virus to nonhuman primates (NHPs) without direct contact [12]. Considering NHPs as surrogates, these findings suggest that pigs could also serve a role in EBOV transmission to humans.

The finding of an ebolavirus in animals, such as domestic pigs, used in intensive production systems and as a major source of human protein is alarming and a cause for public health concern [9, 13]. Serosurveillance studies have provided multiple reports of ebolavirus and ebolavirus-like antibodies being detected in domestic pigs in China [14] and in both East and West Africa, including Uganda [8], Sierra Leone [15], and Guinea [16]. As increasing pork consumption is driving the rapid expansion of pig rearing in areas of sub-Saharan Africa, there is thought to be increased risk of zoonotic pathogen spillover and transmission along the pig–wildlife–human interface [17, 18]. This is especially true in Uganda where pig keeping is actively growing as an increasingly important small stakeholder livelihood strategy [17, 19]. Intriguingly, outbreaks involving ebolaviruses in humans have shown a correlation with periods of peak pork consumption, noting that evidence of increasing demand around holidays may be over represented with a bias due to the occurrence of large family gatherings and higher rates of travel-related activities that could also contribute to the emergence and establishment of an outbreak [15, 18]. Therefore, the risks to both human and animal health associated with the presence of ebolaviruses in livestock produced for human consumption are unknown.

The susceptibility of domestic pigs to ebolaviruses other than RESTV and EBOV, the potential role pigs may play in virus ecology, spillover dynamics, and human public health risk, and the viral shedding and pathogenesis of these viruses in pigs is unknown. For these reasons, we conducted a series of studies aimed at determining the susceptibility of domestic pigs to BDBV. These data are urgently needed to assess the risk this potential host–pathogen interaction could contribute to animal health, food security, and human public health. Here, we demonstrate that domestic pigs are susceptible to experimental infection with BDBV via oronasal inoculation. Infection results in a primary tissue tropism to respiratory and lymphatic tissues leading to the development of moderate interstitial pneumonia and generalized lymphadenopathy. Shedding of infectious virus by the oral and nasal routes was also noted in otherwise subclinical animals.

## 2. Materials and Methods

### 2.1. Biosafety and Animal Ethics

All infectious work and sample inactivation were performed in the Containment Level 4 (CL4) laboratory in accordance with the policies and protocols outlined by the Canadian Science Centre for Human and Animal Health Institutional Biosafety Committee. All animal work was performed in strict accordance with the Canadian Council for Animal Care under the approval and oversight of the facility Animal Care and Use Committee (ACUC). As pigs are social animals, they were group housed throughout the study, under controlled conditions of humidity, temperature, and light (12-hr light/12-hr dark cycles). Food and water were available ad libitum. Animals were fed a commercial pig diet, as well as fruits and vegetables as enrichment, and monitored at least once daily by trained personnel. Environmental enrichment was provided as novel toys and a small pool. Humane endpoints were approved by the ACUC and specified when animals should be humanely euthanized.

### 2.2. Experimental Animal Inoculation

**Figure 1:**
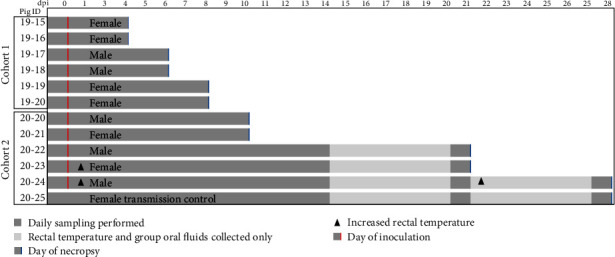
Study design and sample collection timeline for the experimental infection of pigs with BDBV. Two cohorts of pigs were oronasally inoculated with 1 × 10^6^ pfu/mL of Bundibugyo virus (BDBV) under general anesthesia at 0 days-post-inoculation (dpi). Daily sampling was performed at times indicated and included rectal, oral, and nasal swabs; nasal wash; and blood collection. A cursory physical exam, rectal thermometry, and aural pulse oximetry were performed on each animal while under anesthesia at these sampling time points. Individual rectal temperature and collection of oral fluid via group rope chew was performed daily for the duration of the study. The “transmission control,” pig 20-25, was sham inoculated with media at 0 dpi. Animals were observed at least twice daily for development of clinical disease. Two pigs, both in Cohort 2, had slightly elevated temperatures noted at 1 dpi (indicated by filled triangle; 20–23, 40.3; 20–24, 40.5 C). One of those two animals also had an increased temperature at 22 dpi (20–24, 40.4 C). dpi: day post-inoculation; BDBV, Bundibugyo virus.

A total of 14, high-health status, American Yorkshire–Landrace cross, domestic pigs (*Sus scrofa domesticus*) (six castrated males and eight females, age 6 weeks) were used, with seven pigs being used in each of two studies conducted (Figure 1). Pigs were acquired from a mycoplasma—and porcine reproductive and respiratory syndrome virus—certified free herd and were previously vaccinated for porcine circovirus-2 (per vendor provided health records). One pig was sampled and necropsied prior to infection in each study to serve as a farm control. After a 7-day acclimation, the remaining six pigs were oronasally challenged with approximately 1 × 10^6^ pfu total of BDBV inoculum in 3.0 mL media distributed as 1.0 mL per nostril and 1.0 mL placed in the distal pharynx utilizing a sterile tomcat-style catheter. The challenge dose was confirmed by back titration of the inoculum on Vero E6 cells. A physical examination including collection of blood, multiple swabs (rectal, oral, and nasal), and nasal wash was performed at the time points shown in Figure 1. Fluid from group rope chews was collected daily using a commercially available collection kit (Tego Swine Oral Fluids Kit, A100930, ITL BioMedical Animal Healthcare). Extensive tissue sampling occurred at necropsy, including performing a bronchoalveolar lavage (BAL). Animal numbers were not based on power analysis but on the limitations of the maximum containment animal room size. Group assignment (day of euthanasia and necropsy) was based on randomization at the time of permanent animal identification application (ear tag).

### 2.3. Virus Stock and Inoculum Preparation

Stock virus was produced and titrated by an immunocytochemistry assay (described below) on Vero 76 Clone E6 cells in Dulbecco's modified Eagle's medium (DMEM) supplemented with 2% fetal bovine serum (FBS) and stored at −150°C. Viral stocks were diluted to challenge dose in DMEM. The cell line was purchased from ATCC (CRL-1586, Lot 58027482) and confirmed to be bacteria and *Mycoplasma spp*. free. The identity and purity of the isolate used in this study were verified by whole-genome sequencing and confirmed to be BDBV with a 99.33% genome-wide nucleotide identity with *Ebola virus/H. sapiens-tc/Uganda/2007/Bundibugyo-R4386L* (GenBank MK028834).

### 2.4. Virus Titration by Immunocytochemistry

Virus was titrated using a previously described immunocytochemistry assay on Vero E6 cells [20]. Briefly, samples were serially diluted (1 : 10) and incubated on confluent monolayers of cells in 96 well plates at 37°C and 5% CO_2_ for 1 hr. Here, 1.75% of carboxymethylcellulose (CMC; Sigma) containing DMEM, 7.5% bovine serum albumin fraction V, 7.5% sodium bicarbonate, 4-(2-hydroxyethyl)-1-piperazineethanesulfonic acid (HEPES) 1 M, 0.4 g/L 100x folic acid, 200 mM L-glutamine, 11.0 mg/mL sodium pyruvate, and 100x penicillin/streptomycin was added to each well. The plates were incubated for 4 days at 37°C and 5% CO_2_ and then fixed for 24 hr with 10% buffered formalin. The CMC overlay and formalin were removed and 3% Triton X-100 was used to permeabilize the cell monolayer after washing. A primary anti-BDBV nucleoprotein antibody (IBT Bioservices; 0304-001) was added at a dilution of 1 : 2,000 and the plates were incubated at room temperature with agitation. After incubation, the plates were washed with TBS-T and deionized water and an antirabbit secondary antibody was applied as per manufacturer's instructions (Dako; K4011). Following room temperature incubation and washing with TBS-T and water, substrate was added (Dako EnVision+ Substrate System; K3468). The plates were washed with deionized water and allowed to dry before the stained plaques were counted with the aid of a stereo dissecting microscope.

### 2.5. Sampling of Animals

Oral, rectal, and nasal swabs were taken from each pig at the time points outlined in Figure 1 under general anesthesia using isoflurane and placed into sterile Dulbecco's phosphate-buffered saline (D-PBS) containing the following antibiotics: streptomycin, vancomycin, nystatin, and gentamycin. Fluid was collected from a bilateral nasal wash with sterile D-PBS and one of each of the following tubes was collected via jugular venipuncture: serum, sodium citrate, sodium heparin, and K3 ethylenediaminetetraacetic acid (EDTA) Figure (1). While under anesthesia, a physical exam including rectal temperature and pulse oximetry (Massimo Rad 5) was conducted on each pig.

### 2.6. Complete Blood Counts, Blood Chemistry, and Blood Gas Analyses

Hematology was performed on a VetScan HM5 hematology analyzer (Zoetis, USA) using K3 EDTA-treated whole blood, and the following parameters were evaluated: red blood cells, hemoglobin, hematocrit, mean corpuscular volume, mean corpuscular hemoglobin, mean corpuscular hemoglobin concentration, red cell distribution weight, platelets, mean platelet volume, white blood cells, neutrophil count (absolute (abs) and %), lymphocyte count (abs and %), monocyte count (abs and %), eosinophil count (abs and %), and basophil count (abs and %). Blood chemistries were evaluated on a VetScan VS2 (Zoetis, USA) with the Preventative Care Profile Plus rotor (Zoetis) using sodium heparin-treated whole blood and the following parameters were evaluated: glucose, blood urea nitrogen, creatinine, calcium, albumin, total protein, alanine aminotransferase, aspartate aminotransferase, alkaline phosphatase, total carbon dioxide, potassium, sodium, chloride, globulin, and total bilirubin. Sodium heparin-treated blood was also used to analyze blood gases, which were performed on an iSTAT Alinity V machine (Zoetis, USA) using a CG4+ cartridge (Zoetis, USA) to measure the following parameters: lactate, pH, total carbon dioxide, partial pressure carbon dioxide, partial pressure oxygen, soluble oxygen, bicarbonate, and base excess. All analyses were performed according to the manufacturer's instructions.

### 2.7. Necropsy and Tissue Sampling

Necropsies were performed after euthanasia via pentobarbital overdose, confirmation of death, and exsanguination by femoral artery laceration. The following tissues were collected and individual samples split between 10% neutral-buffered formalin and fresh tissue: cerebrospinal fluid, urine, vitreous fluid, skin, skeletal muscle, abdominal fat, liver, spleen, pancreas, duodenum, jejunum, ileum, spiral colon, kidney, gastrohepatic and mesenteric lymph nodes, right cranial lung lobe, right middle lung lobe, right caudal lung lobe, left cranial lung lobe, left caudal lung lobe, trachea, heart, tracheobronchial lymph nodes, cervical spinal cord, meninges, cerebrum, cerebellum, brainstem, olfactory bulb, nasal turbinates, submandibular lymph nodes, tonsil, trigeminal ganglion, and the entire eye. The superficial inguinal, caudal cervical, and iliac/sublumbar lymph nodes were also collected. The reproductive tracts (uterus and ovaries) were collected *en bloc* from female animals.

### 2.8. Histopathology

Histopathology, immunohistochemistry, and *in situ* hybridization (ISH) were performed based on positive results from reverse transcriptase polymerase chain reaction (rRT-PCR). After fixation and inactivation of tissues in 10% neutral phosphate buffered formalin for a minimum of 7 days, tissues were trimmed and removed from the maximum containment laboratory, routinely processed, and stained with Gill's hematoxylin and eosin (HE) for histopathologic examination.

### 2.9. In Situ Hybridization for Detection of BDBV in Tissue Sections

ISH was utilized to detect BDBV genomic material in paraffin-embedded formalin-fixed tissue sections. Five micrometers paraffin-embedded formalin-fixed tissue sections were cut, air dried, and then melted on to charged slides in an oven at 60°C. The slides were then cleared and dehydrated in xylene and 100% ethanol and then air dried. The sections were quenched in aqueous H_2_O_2 for 10 min_, boiled in target retrieval solution for 15 min, rinsed in 100% ethanol, and air dried again. Then, a final pretreatment of protease plus enzyme for 15 min at 40°C was applied. The probe (V-BDBV-NP-C1; Advanced Cell Diagnostics) was applied and incubated at 40°C for 2 hr. The hybridization amplification steps (AMP 1–6) were applied to the slides for the recommended times and temperatures as per the manual for the RNAscope® 2.5HD Detection Reagent—Red kit (Advanced Cell Diagnostics). The signal was then visualized by the chromogen Fast Red. Sections were then counter stained with Gill's 1 hematoxylin, dried, and cover slipped.

### 2.10. Combination Immunohistochemistry and In Situ Hybridization Staining

A combination of immunohistochemistry (IHC) and ISH staining was carried out for the identification of BDBV infected macrophages and pneumocytes in lung tissue sections. Five micrometers paraffin-embedded formalin-fixed tissue sections were cut, air dried, and then melted on to charged slides in an oven at 60°C. The slides were then cleared and dehydrated in xylene and 100% ethanol and allowed to air dry. Sections were quenched for 10 min in aqueous hydrogen peroxide.

For identification of infected macrophages, epitopes were retrieved using an AR10 target retrieval kit (Biogenex, USA) in a BioCare Medical Decloaking Chamber. Mac387, a mouse antihuman macrophage monoclonal antibody (MCA874G; BioRad, USA), was used at a 1 : 50 dilution at 4°C overnight in a humidified chamber. Slides were then fixed with 10% neutral buffered formalin, rinsed, and followed by a final pretreatment of protease plus enzyme for 15 min at 40°C. The BDBV-specific ISH probe (V-BDBV-NP-C1, Advanced Cell Diagnostics) was applied and incubated at 40°C for 2 hr. RNAscope® 2.5HD Detection Reagent—Red kit (Advanced Cell Diagnostics) hybridization amplification steps (AMP 1–6) were followed as per the kit instructions. The signal was then visualized by the chromogen Fast Red. Slides were treated with codetection blocker (Advanced Cell Diagnostics); and the IHC process was completed using a horse radish peroxidase labelled polymer and Envision® + system (antimouse) (Agilent Technologies, USA), followed by reaction with the chromogen diaminobenzidine. The sections were then counter stained with Gill's 1 hematoxylin, dried, and cover slipped.

For the identification of infected pneumocytes, epitopes were retrieved using Co-Detection Target Retrieval (Advanced Cell Diagnostics, pH 9-10) for 15 min. A 1 : 50 dilution of a mouse antihuman cytokeratin monoclonal antibody (clonesAE1/AE3, Agilent) was adsorbed overnight in a humidified chamber at 4°C. The processes described for infected macrophage identification were followed, beginning with fixation of the slides with 10% neutral buffered formalin.

### 2.11. RNA Detection by Quantitative Real-Time PCR

The following samples were tested for the presence of viral RNA by real-time rRT-PCR targeting the BDBV *L* gene: citrated whole blood, nasal swabs, rectal swabs, oral swabs, nasal wash, urine, vitreous, cerebrospinal fluid, BAL fluid (BALF), and group rope chew fluid. Fresh tissues collected at necropsy were cut into 0.1–0.5 g portions, distributed into tissue grinding tubes (KT03961; Precellys Lysing Kit, USA), and immediately frozen at −70°C for further processing.

Tubes were later thawed, D-PBS added to 10%, and homogenization proceeded using a bead mill homogenizer for a minimum of 45 s at the maximum setting. Each tube was centrifuged at 1,500x *g* for 20 min at 4°C prior to sampling. Tissues, all swabs, BALF supernatant, BALF cell pellet, nasal wash fluid, cerebrospinal fluid (CSF), vitreous, and urine were inactivated as a 1 : 10 suspension in Tripure Reagent, removed from the CL4 laboratory, and stored at −70°C until further processing. Citrated blood samples were inactivated with TriZol LS as per the manufacturer's instructions prior to removal from the CL4 laboratory.

The MagMax CORE Nucleic Acid Purification Kit (Applied Biosystems, A73202) was utilized for the extraction of total RNA from inactivated samples as per manufacturer's recommendation with some modification. Tripure Reagent or TriZol LS was utilized in place of the manufacturer's lysis buffer for inactivation and removal of BDBV RNA from the CL4 laboratory. Six hundred fifty microlitres of inactivated sample and 350 *µ*L of kit-provided core binding buffer were utilized followed by a final elution volume of 30 *μ*L using the automated MagMax Express 96 system (ThermoFisher Scientific). Enteroviral armoured RNA (ARM-ENTERO; Asuragen) was utilized as an exogenous extraction and reaction control. A standard curve was generated by serial tenfold dilutions of a BDBV plasmid provided by the Public Health Agency of Canada and included in every run allowing for the semiquantification of viral load, measured in copies/reaction. This plasmid contains the coding region of the BDBV *L* gene and the pCAGGS backbone derived from the same isolate used as the inoculum in this study. In addition, tripure-inactivated cell culture-derived BDBV stock virus was utilized in each run as a positive control. Extracted product was stored at −70°C until real-time rRT-PCR was performed using a 4x TaqMan FAST Virus 1 Step kit, in a reaction volume of 20 *μ*L using the following primer pair and probe combinations and concentrations: BDBV: 0.4 *µ*M BDBV L-gene forward 1 : 5′-CCGAGAAAATCCACCAGAAG−3′, 0.4 *µ*M BDBV L-gene reverse: 5′-TGTTGRAGTCCCTCAATYCC−3′, and 0.1 *µ*M BDBV L-gene probe: 5′-YCCAAGCTCTTACCGTGGTCATCTTGG−3; and ARM-ENTERO: 0.2 *µ*M ARM-ENTERO−31 forward: 5′-ATG CGG CTA ATC CCA ACCT−3′, 0.2 *µ*M ARM-ENTERO−31 reverse: 5′-CGT TAC GAC AGG CCA ATC ACT−3′, and 0.06 *µ*M ARM-ENTERO−31 probe: 5′-CAG GTG GTC ACA AAC−3′.

Reactions were run on a 7500 Fast machine (Applied Biosystems) with the following conditions: 50°C for 5 min, 95°C for 20 s, and 40 cycles of 95°C for 3 s followed by 60°C for 30 s. Cycle threshold (Ct) values were determined by the 7500 Fast software and the respective virus gene copy numbers were calculated for each sample, based on Ct values for each respective standard curve. All samples were assayed as technical replicates.

Sample baselines were determined by the default settings of the ABI 7500 software. The Ct value threshold was determined by separate analysis of the standard curve, and then the threshold applied to the remainder of the samples to determine the Ct values.

### 2.12. Virus Isolation from Samples and Tissues

Isolation was conducted on Vero E6 cells plated in a 48-well format at 80% confluence. Media were removed and monolayers were washed with D-PBS prior to 1 : 10 and 1 : 100 dilutions of samples in DMEM being adsorbed in duplicate to the monolayers for 1 hr at 37°C and 5% CO_2_. Tissues were homogenized as described for RNA detection and diluted in DMEM from 1 : 10 to 1 : 100,000 and adsorbed to washed monolayers. DMEM with 2% FBS, penicillin/streptomycin, and L-glutamine was then added to each well and plates were returned to humidified incubation at 37°C and 5% CO_2_. All plates were read for cytopathic effect at day 14 for evidence of virus. Samples were assayed in duplicate and the sample origin was blinded until after results were recorded. Duplicate negative controls (DMEM) and positive controls (challenge virus stock) were included on each test plate.

### 2.13. Antibody Analyses

BDBV-specific IgM and IgG were measured in serum by enzyme-linked immunosorbent assay (ELISA) using commercially available recombinant BDBV glycoprotein without the transmembrane component (rBDBV GPdTM) (IBT Bioservices; 0505-015) and peroxidase-labeled rabbit antipig IgG (polyclonal, H + L, 1 : 1,000 dilution; Invitrogen; PA1-28602) or goat antipig IgM (polyclonal, 1 : 5,000 dilution; BioRad laboratories; AA148) with a two-step substrate system (KPL 2,2'-azino-di-(3-ethylbenzthiazoline-6-sulfonic acid) (ABTS) Peroxidase Substrate System; SeraCare). BDBV-specific IgA was measured in nasal wash fluid or oral fluid collected by rope chew using an adaptation of the IgM and IgG protocol. The same rBDBV GPdTM antigen was diluted in PBS (1 : 5,000) and used to coat Nunc MaxiSorp F plates at 0.5 *µ*g/mL. Five percent skim milk and 0.5% Tween-20 in PBS were used as both a washing and blocking buffer, respectively. Serial twofold dilutions made in blocking buffer were assayed in duplicate beginning at a 1 : 4 dilution until the end point. Bound antibody was detected with peroxidase-labeled goat antipig IgA (polyclonal, 1 : 5,000 dilution; BioRad Laboratories; AA140) followed by the ABTS substrate described for the IgM and IgG ELISAs. Absorbance for all three ELISAs was measured at 405 nm using a plate reader (BioTek Epoch microplate spectrophotometer with Gen5 Data Analysis software, version 1.11). Sera with an OD greater than the average negative OD plus three standard deviations were considered positive.

### 2.14. Viral Neutralization Assay

Neutralization activity of all serum collected during the study was determined by a modified plaque reduction neutralization test against BDBV. Serial twofold dilutions of heat inactivated (56°C for 30 min) sera were incubated with virus for 1 hr at 37°C. Each mixture of serum and virus was then added to duplicate wells containing confluent monolayers of Vero E6 cells in a 96-well format, incubated for 1 hr at 37°C, and overlaid with 150 *μ*L of 1.75% CMC in DMEM per well. Plates were then incubated at 37°C and 5% CO_2_ for 72 hr and fixed with 10% buffered formalin. Virus not neutralized by the serum was measured by immunocytochemistry as described for the titration of virus. Serum dilutions resulting in >70% reduction of plaque counts compared with virus controls were considered to be positive for virus neutralizing activity. Serum from each animal was screened for non-virus-specific neutralizing activity against assay components [21].

### 2.15. Genome Sequencing

Challenge virus stock used as the inoculum for both cohorts one and two and, based on results from rRT-PCR, the homogenized tissue samples collected from each animal were evaluated by whole-genome sequencing. A database of all publicly available *Bundibugyo ebolavirus* complete genomes was downloaded from NCBI on June 7, 2021. Genomes were aligned with MAFFT v7.48 on default settings, and the resulting alignment was inspected within Geneious v9.1.8 to confirm that maximum sequence divergence did not exceed 5%, as per the instructions of the Primal Scheme website [22, 23]. In addition, Primal Scheme recommends that genomes with 99% or higher identity be removed to reduce bias during primer selection. To remove genomes with high similarity, CD-HIT-EST web server was used with a sequence identity cut-off of 0.99 [24]. The resulting database was used to generate PCR primers for tiled whole-genome amplification using the Primal Scheme web server [25]. Designed primers were manually compared to the previously generated whole genome database and inspected using Geneious v9.1.8 to look for mismatches within primers that could potentially impact amplification efficiency [23]. Based on this manual inspection, it was determined that the scheme with an amplicon size of 800 bp yielded the best primers.

Reverse transcription was performed on extracted nucleic acid using the LunaScript RT SuperMix Kit (New England Biolabs Inc.) according to the manufacturer's instructions. PCR amplification was performed using the previously designed primers and Q5 High-Fidelity 2x Master Mix (New England Biolabs Inc.) according to the conditions used by Quick et al. [25], except with 32 amplification cycles. Here, overlapping amplicons tiling across the genome were generated. To prevent the generation of unwanted PCR products, two separate reactions were generated for each sample containing primers for alternating PCR products. Following amplification, the two reactions for each sample were combined, cleaned using AMPure XP (Beckman Coulter) size selection beads at 1x concentration, and quantified using Qubit 1x dsDNA HS Assay Kit on the Qubit 3.0 Fluorometer (Thermo Fisher Scientific). Sequencing libraries were generated using the Nextera XT library preparation kit (Illumina) as per manufacturer's instructions. Libraries were quantified, pooled, and sequenced on the Illumina MiSeq using a V3 flow cell and a 600 cycle kit (Illumina).

## 3. Results

### 3.1. Clinical Features

Seven-week-old pigs were challenged oronasally with 1.0 × 10^6^ pfu to determine the susceptibility of domestic swine to BDBV. Two animals, both in the second cohort, had mildly increased rectal temperatures at 1-day post-inoculation (dpi) (40.3 and 40.5°C). One of these two animals also had a mildly increased temperature at 22 dpi (40.4°C). Temperature response at all other time points for these animals and all time points for the other pigs in both cohorts remained within range (≤40.0°C) for the duration of the study (Figure 1 and *Supplementary 1*). No additional clinical manifestations were observed in either the inoculated animals or the sham-inoculated transmission control pig. Review of pulse oximetry and clinical pathology data, including complete blood counts, blood chemistries, and blood gas analyses, revealed no significant findings (*Supplementary 1*).

### 3.2. Detection of BDBV RNA and Infectious Virus in Serial Samples

Swabs (oral, nasal, and rectal), nasal wash fluid, and blood were collected to detect and quantify shedding of virus following inoculation (Table 1 and *Supplementary 1*). Genomic material was extracted from all samples and rRT-PCR was performed to detect the presence of BDBV by targeting the *L* gene, a component of the viral RNA-dependent RNA polymerase. Following rRT-PCR, virus isolation was performed on all positive samples. BDBV RNA was readily detected in the nasal swab samples from inoculated pigs beginning at 1 to 5 dpi with the latest detection in two of seven remaining animals at 7 dpi. The peak level of 9.5 × 10^5^ copies/mL, occurred at 5 dpi. Of the 53 total rRT-PCR positive nasal swab samples collected, infectious virus was successfully recovered from a total of 15 samples, at least once from 8 of the 11 inoculated pigs, ranging from 1 to 6 dpi (Table 1 and *Supplementary 1*). Five inoculated animals had rectal swabs test positive by rRT-PCR at either 1 or 2 dpi, but infectious virus was not recovered from any of these samples (Table 1 and *Supplementary 1*). BDBV RNA was not detected in the blood at any time point in the study (Table 1 and *Supplementary 1*).

Nasal wash fluid is considered as a sensitive method for pathogen detection in swine. We deployed this sampling technique using sterile D-PBS to rinse nasal passages, with recovered fluid evaluated for the presence of BDBV RNA by rRT-PCR and infectious virus by viral isolation. In addition, this sampling method and matrix was used to monitor the development of IgA antibody. All inoculated animals were rRT-PCR positive at least once, ranging from 1 to 10 dpi, with a peak level of 4.8 × 10^7^ copies/mL occurring at 1 dpi. As this could represent residual inoculum, it is also noted that the next highest viral load occurred at 5dpi (1.4 × 10^6^ copies/mL). Infectious virus was recovered from 10 of the 11 animals in a total of 24 nasal wash samples ranging from 1 to 9 dpi (Table 1 and *Supplementary 1*).

Collection of oral fluids by rope chew was investigated as a potential noninvasive sampling method for evaluating BDBV shedding at the group level. To accomplish this, a cotton rope was hung in the animal pens at pig shoulder height providing opportunity for animals to interact with the item for 20–30 min. Oral fluids deposited on the rope were collected and processed daily throughout the study. This method of sampling proved effective, with the presence of BDBV RNA successfully detected by rRT-PCR from 1 to 8 dpi and infectious virus recovered on 2, 3, 5, and 6 dpi (Table 2 and *Supplementary 1*).

### 3.3. Gross Pathology and Viral Tissue Distribution

**Figure 2:**
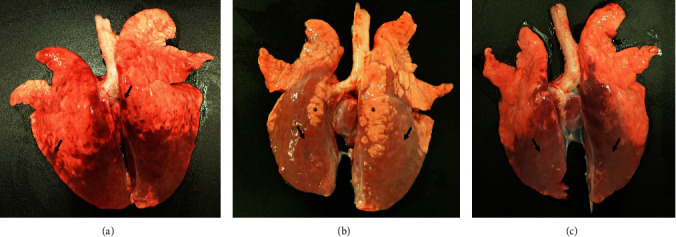
Gross lung pathology of Bundibugyo virus infected pigs. (a) At 4 days postinfection (dpi), lungs were congested ( ^*∗*^) with scattered hemorrhagic lobules (arrows). (b) At 6 dpi, the dorsal aspect of the caudal lung lobes had locally extensive areas that were firm, depressed, and reddened indicating areas of pneumonia (arrows) surrounded by areas of normal lung tissue ( ^*∗*^). (c) These lesions were also present at 8 dpi.

**Figure 3:**
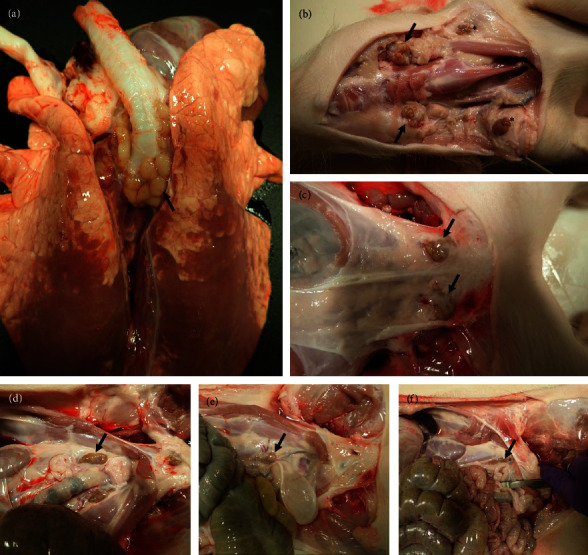
Lymphadenopathy in Bundibugyo virus infected pigs. A generalized lymphadenopathy was noted during gross examination with lymph nodes appearing diffusely firm and tan to tan-dark red and which appeared to bulge on cut section. Lymph nodes from numerous anatomic locations were involved, including the (a) tracheobronchial, (b) mandibular (arrow) and retropharyngeal ( ^*∗*^), and (c) superficial inguinal nodes. The iliac and sublumbar lymph nodes were also involved at (d) 4, (e) 6, and (f) 8 days post-inoculation.

**Figure 4:**
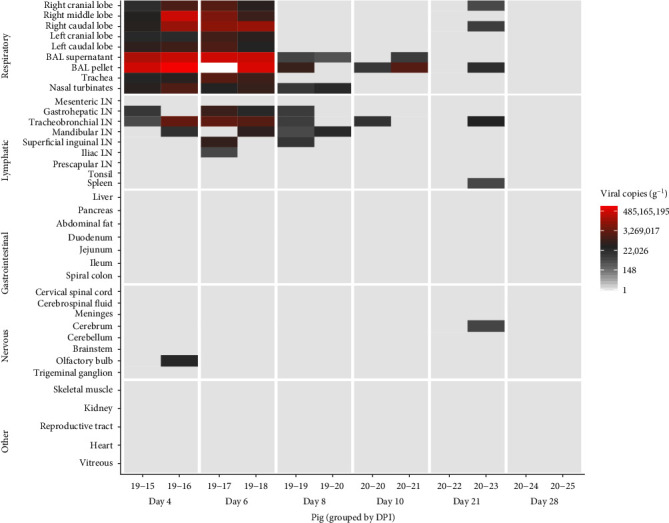
Distribution and quantification of BDBV in tissues of experimentally infected pigs. The heatmap depicts the mean of technical replicates as BDBV copies per gram of tissue as determined by rRT-PCR by day post-inoculation (dpi). Tissues are grouped by general anatomic system showing that BDBV predominately affects the respiratory and lymphatic tissues in the pig. Tissues for which infectious virus was successfully isolated are indicated (+). BAL, bronchoalveolar lavage.

To identify the tissue tropism and gross lesions secondary to infection with BDBV, we performed necropsies on two animals each at 4, 6, 8, 10, 21, and 28 dpi (Figure 1). The overall gross and histopathology findings are indicative of the development of a moderate interstitial pneumonia with a generalized lymphadenopathy by 6 dpi (Figures 2 and 3). Tissue viral RNA load, as determined by rRT-PCR and virus isolation, is concurrent with the pathology findings, demonstrating a predominately respiratory and lymphatic tissue involvement (Figure 4).

Gross lesions observed during necropsy were limited to the lungs and lymph nodes. At 4 dpi, pulmonary congestion with scattered hemorrhagic lobules were noted primarily involving the caudal lung lobes (Figure 2(a)). At 6 dpi, the dorsal aspect of the caudal lobes appeared diffusely reddened, firm, and depressed consistent with the presence of a moderate interstitial pneumonia (Figure 2(b)). Similar findings were noted in animals necropsied at 8 dpi (Figure 2(c)). At 10, 21, and 28 dpi, there were areas of reddening present on the dorsal aspect of the caudal lung lobes, but these areas were not depressed or firm. We suspect that this is most likely artifactual congestion secondary to euthanasia (*Supplementary 2*). A generalized lymphadenopathy was present during gross examination at necropsy with moderate to severe enlargement of the tracheobronchial, mandibular, and retropharyngeal lymph nodes noted at 6, 8, and 10 dpi (Figures 3(a) and 3(b)). These lymph nodes were diffusely firm, tan-dark red, and bulged on cut section. The superficial inguinal and mesenteric lymph nodes were also moderately enlarged during these early time points (Figure 3(c)). The iliac and sublumbar lymph nodes were observed to be variably enlarged from 4 to 21 dpi (Figure 3(d)–3(f)).

The rRT-PCR was performed across all tissues and samples collected at necropsy with subsequent virus isolation carried out for samples testing positive (Figure 4). These results indicate a predominant involvement of the respiratory tissues with viral RNA detected in the lungs, trachea, and nasal turbinate tissues, as well as the BAL samples, until 21 dpi with peak viral RNA loads reached at 4 and 6 dpi. BDBV RNA was regularly detected in tracheobronchial, mandibular, and gastrohepatic lymph nodes, as well as sporadically detected in the superficial inguinal, iliac, and sublumbar lymph nodes. Low levels (1.4 × 10^3^ copies/mL) of virus were detected in the spleen of only one animal that was necropsied at 21 dpi. The olfactory bulb of one animal and the cerebrum of another animal had low levels of detectable virus at 4 and 21 dpi, respectively. Infectious virus was successfully isolated from a variety of respiratory and lymphatic tissues between 4 and 21 dpi, including from lung, trachea, nasal turbinate, BAL, and lymph node samples (Figure 4). Neither RNA nor infectious virus were detected in tissue samples after 21 dpi.

### 3.4. Histopathology and In Situ Hybridization

**Figure 5:**
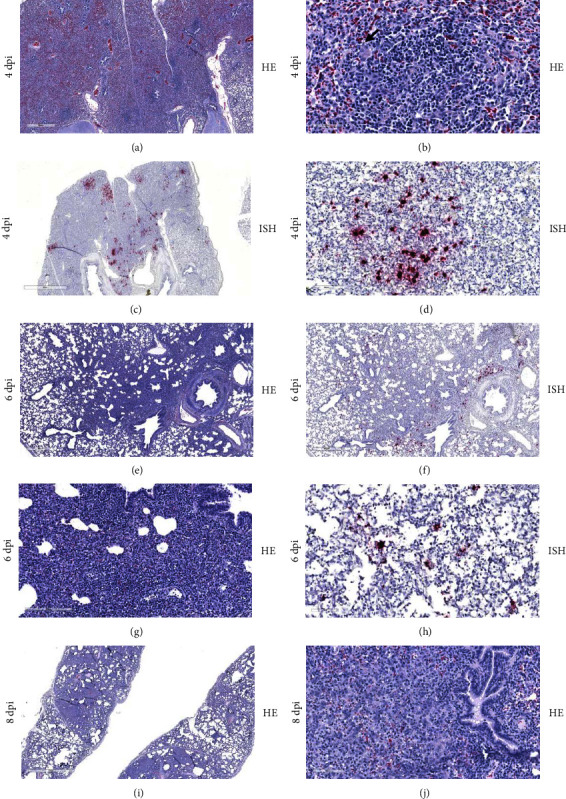
Lung histopathology and in situ hybridization of Bundibugyo virus infection in pigs. Histopathologic lesions of BDBV infection of the lungs were consistent with the development of moderate to severe interstitial pneumonia. (a) At 4 dpi, an extensive infiltration of inflammatory cells was noted resulting in the loss of air space. (b) This infiltrate appeared to consist predominately of lymphocytes and macrophages with scattered multinucleated cells consistent with syncytia formation (arrow). (c) Viral RNA was detected by ISH in areas corresponding to sites of inflammation and (d) as well as being found within macrophages. (e, f) Similar lesions were observed at 6 dpi and (g, h) also corresponded to the detection of viral RNA. (i) Occasional peri-bronchiolar inflammation was detected at 8 dpi, but (j) the presence of type II pneumocyte hyperplasia is indicative of transition to the reparative phase in these lesions. ISH, in situ hybridization; HE, hematoxylin and eosin stain; dpi, day post-inoculation; BDBV, Bundibugyo virus.

**Figure 6:**
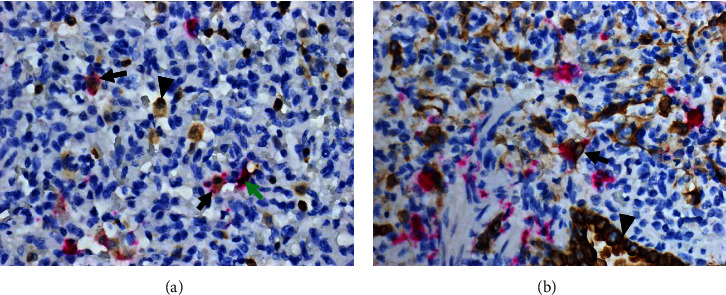
(a, b) Pulmonary macrophages and pneumocytes are infected during Bundibugyo virus infection in domestic pigs. A combination of immunohistochemistry (IHC) and in situ hybridization (ISH) techniques were used to identify Bundibugyo virus (BDBV) infection of cell types in the lung tissue of experimentally infected pigs. Macrophages (stained brown; a, arrowhead) were identified by IHC and infected cells (stained pink; a, green arrow) were identified by ISH. Scattered infected macrophages were observed in areas of lesions (stained brown and pink; a, black arrow). Epithelial cells (stained brown; b, arrowhead) were identified by IHC and infected cells (stained pink; b, grey arrow) were identified by ISH. Numerous infected pneumocytes (stained brown and pink; b, black arrow) were identified throughout the lung section.

Histopathologic evaluation revealed lung lesions of varying degrees of severity observed from 4 to 21 dpi. At 4 dpi, congestion, atelectasis, peri-vasculitis, peri-bronchiolitis, and interlobular edema were noted. Several lung sections showed extensive infiltration of inflammatory cells resulting in the loss of air spaces (Figure 5(a)). These cells appeared to be predominately lymphocytes and macrophages with the scattered presence of multinucleated cells consistent with the formation of syncytia (Figure 5(b)). ISH detected viral RNA within these areas of inflammation, primarily located within macrophages (Figures 5(c) and 5(d)). Viral RNA was also detected within pneumocytes. A combination of IHC and ISH staining was utilized to confirm the presence of BDBV genomic material within both pulmonary macrophages (Figure 6(a)) and pneumocytes (Figure 6(b)). Similar histopathologic pulmonary lesions were observed at 6 dpi (Figures 5(e) and 5(f)) and were similarly correlated with the presence of viral antigen detected by ISH (Figures 5(g) and 5(h)). These lesions are consistent with moderate multifocal interstitial pneumonia with peri-bronchiolar inflammation and were present through 8 dpi (Figure 5(i)). Type II pneumocyte hyperplasia was noted at 8 dpi, indicating that the lung lesions were in the reparative phase (Figure 5(j)). At 10 dpi, lung lesions consisted of mild to moderate interstitial infiltrates with the presence of inflammatory cells causing the expansion of alveolar septa. The associated pneumonia was less severe compared to earlier time points and alveolar spaces were no longer seen to be obliterated (*Supplementary 2*). There were multifocal areas of interstitial infiltration of lymphocytes and macrophages still present at 21 and 28 dpi, but the lesions were mild (*Supplementary 2*). Viral RNA was not detected by ISH in lung tissue after 6 dpi.

**Figure 7:**
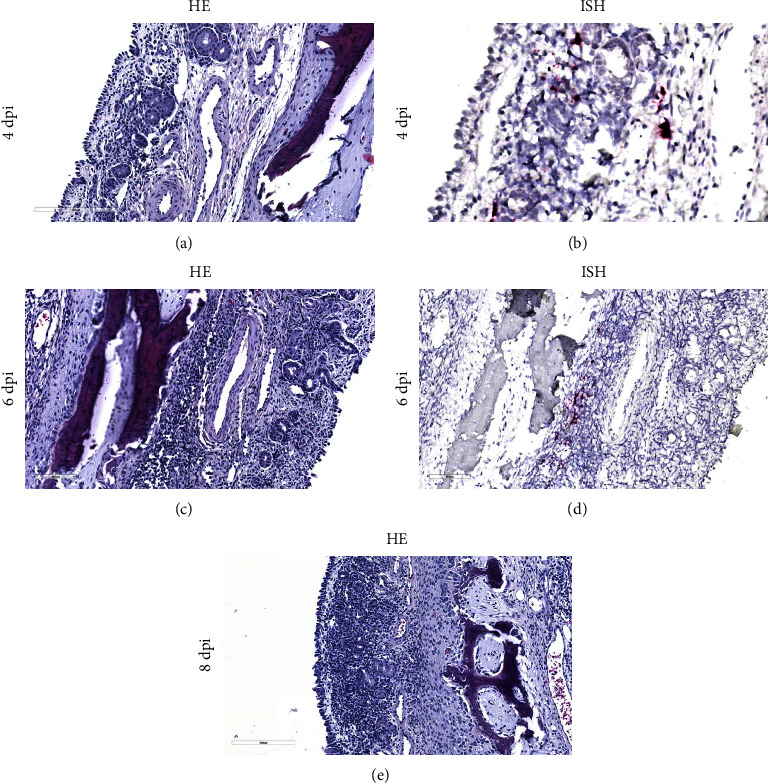
Nasal turbinate histopathology and *in situ* hybridization of Bundibugyo virus in pigs. Evaluation of the nasal turbinates in BDBV infected pigs showed the presence of multifocal areas of submucosal inflammation at both (a) 4 (a) and (b) 6 dpi. (c, d) These areas of inflammation corresponded to the detection of viral RNA by ISH. (e) At 8 dpi, submucosal inflammation was accompanied by multifocal degeneration and loss of surface epithelial cells. ISH, *in situ* hybridization; HE, hematoxylin and eosin stain; dpi, day postinfection; BDBV, Bundibugyo virus.

Evaluation of the nasal turbinates showed areas of submucosal inflammation at both 4 and 6 dpi (Figures 7(a) and 7(c)). These areas of inflammation corresponded to the presence of viral RNA as detected by ISH (Figures 7(b) and 7(d)). At 8 dpi, inflammation of the submucosa was accompanied by multifocal degeneration and loss of surface epithelial cells (Figure 7(e)), but viral RNA was not detectable in the nasal turbinates at or after this time point. The submucosal inflammation noted at earlier time points was still present at 21 dpi, but no significant lesions were detected by 28 dpi. At all time points, there was evidence of paracortical hyperplasia in lymph nodes, indicative of a reactive state. Histopathologic evaluation revealed no significant lesions in other evaluated tissues.

### 3.5. Evaluation of the Anti-BDBV Antibody Response

The development of a BDBV-specific antibody response was monitored over the course of the study utilizing serial serum, nasal wash fluid, and group oral fluid samples. All individual animals and oral fluid samples from both cohorts were negative for the presence of BDBV-specific antibodies at 0 dpi. IgM and IgG titers were detectable in serum samples of seven inoculated pigs seroconverting at time points from 6 to 11 dpi (Tables 3 and 4, *Supplementary 2*). Both IgM and IgG titers ranged from 1 : 100 to 1 : 1,600, with a titer of 1 : 100 being the result most frequently encountered throughout the study. IgM titers for two animals, pigs 20-22 and 20-24, were first detected at 11 dpi but both animals had no detectable titers at the time of euthanasia (21 and 28 dpi). Both IgM and IgG levels appeared to decrease within the study's timeframe, but only IgG titers were detectable at the time of euthanasia in the inoculated animals.

Six inoculated pigs developed an IgA response detectable in nasal wash fluid between 4 and 16 dpi, with titers ranging from 1 : 4 to 1 : 16 (Table 5). This appears to loosely correspond to the development of detectable IgM and IgG in sera. One exception would be that pig 20-24 seroconverted with both IgM and IgG but not IgA. Group oral fluid samples collected by cotton ropes were assayed for the detection of IgA utilizing the same ELISA approach described for nasal wash samples. For cohort one, IgA was detected only on the day of study termination, 8 dpi, at a titer of 1 : 4. IgA was detected in cohort two samples beginning at 10 dpi and detected daily until 21 dpi, with the exception of a single time point (Table 6).

All serum samples were tested for the presence of a neutralizing antibody response capable of blocking BDBV infectivity in cell culture using a modified plaque reduction neutralization assay approach. Neutralization activity was not detected in any sample at any time point throughout the study. Seroconversion was monitored in the sham-inoculated, transmission control animal, Pig 20-25, in the same manner as the rest of the study animals. IgM was not detected in nasal wash fluid at any time, but IgG antibodies were detected from days 12 to 21 at a constant titer of 1 : 100 (Tables 3 and 4). IgG was not detected at day 28, the time of euthanasia. IgA was detected only at day 13 in nasal wash fluid at a level of 1 : 4 (Table 5). Similar to the inoculated study animals, a neutralizing antibody response was not detected at any time point for the transmission control.

### 3.6. Genomic Evaluation

Utilizing a tiled amplicon approach, virus sequences in tissues revealed limited changes compared to the reference genome and challenge virus stock. Sequencing revealed the challenge virus stock to have a 99.33% genome-wide nucleotide identity with the original isolate, *Ebola virus/H.sapiens-tc/Uganda/2007/Bundibugyo-R4386L* (GenBank MK028834). When the genomes of virus isolated from terminal tissue samples and the inoculum were aligned against the annotated parent genome, a total of four single nucleotide polymorphisms (SNPs) were detected based on the available annotation of the reference isolate (MK028834). Only one SNP was discovered in a coding region, and this was the region designated for Viral Protein 24 (VP24), with the remaining three SNPs found in noncoding regions (*Supplementary 1*). The mutations in noncoding regions were only found in the nasal turbinate samples in two of the sampled animals, and the mutation in the VP24 coding region was found only in the nasal turbinate of a single animal.

## 4. Discussion

Pig production continues to expand to meet the protein demands of a rapidly growing human population. Small stakeholder pig keeping has also become a critical livelihood strategy in many income-restricted regions of the world [8, 17, 19]. Introduction and development of pig production systems in biodiverse landscapes can create new risks for spillover and zoonotic transmission of novel pathogens [17, 18]. Therefore, the continued evaluation of the susceptibility of livestock animals to high risk pathogens, such as the ebolaviruses, is critical to properly informing risk assessments and mitigation strategies for vulnerable regions [26].

The present study demonstrates the first experimental infection study providing evidence that domestic pigs are susceptible to BDBV. Our data indicate that, following mucosal exposure, the development of a productive infection, tissue dissemination, and shedding of infectious virus can occur while animals remain subclinical. The presence of relatively high viral loads in the upper and lower airways suggests that the respiratory mucosa plays an important role during infection in this model and likely contributes to release of virus into the environment. The use of an oronasal route of inoculation was chosen to mimic what is thought to be the most likely route of natural infection while also allowing comparison to previous studies evaluating infection characteristics of other ebolaviruses, RESTV and EBOV, in pigs [11, 12, 27, 28]. Intraspecies transmission of ebolaviruses has been previously documented in pigs, both experimentally with EBOV and naturally with RESTV [9, 12]. Our data also indicate that experimental pig-to-pig transmission may be possible with BDBV, as evidenced by the development of a limited, low-level antibody response in a co-housed, naïve transmission control animal. However, the lack of detectable virus in serial samples or tissues in this animal indicates that further studies should be performed to specifically evaluate intraspecies transmission in swine.

Based on the data reported here and the similarities to studies with RESTV [27, 28] and EBOV [11, 12, 29], we hypothesize that BDBV can infect pigs via the oronasal mucosa, entering the airway epithelium through the apical side, and then replicating to high levels in the pulmonary macrophages, pneumocytes, and endothelial cells. BDBV is then released back into the airway where it can, most likely, be transmitted to naïve pigs through mucosal exposure.

The BDBV isolate utilized in this study originated from the blood of a human case involved in the 2007 outbreak in Uganda [4]. Whole-genome sequencing confirmed that the inoculum used showed a 99.33% genome-wide nucleotide identity matching the original isolate's reference genome. Deep sequencing of the BDBV genome in tissue samples, except for two nasal turbinate samples, revealed no mutations when compared to the challenge virus stock used for inoculation, indicating that these results are likely not secondary to virus mutation, host adaptation, or readily attributable to the selection of quasispecies with different viral fitness, though it must be considered that host adaptation of the ebolaviruses may only require minor changes to the genome. For instance, when comparing wild-type virus to the mouse-adapted strain of the Mayinga variant of EBOV, only 13 nucleotide changes were seen in host adaptation after repeat passage. Only two of these mutations, one in the nucleoprotein and one in VP24, were required to make the wild-type EBOV uniformly lethal in mice [30]. Epidemiological studies of disease caused by ebolaviruses have identified a range of pathogenic phenotypes not linked to specific mutations in the viral genome, suggesting that host response to infection may be responsible for disease severity [31, 32]. This was demonstrated with EBOV by Rasmussen et al. [33] through the use of a collaborative cross panel of recombinant inbred mice showing that genetic factors play a significant role resulting in distinct disease phenotypes ranging from complete resistance to severe disease with 100% lethality.

Although human aspects of emergency response must remain a priority, future sampling of livestock in both geographic and temporal proximity to outbreaks involving ebolaviruses will greatly aid in further refining risk assessments. Our findings suggest that future sampling initiatives in pigs should consider the inclusion of nasal and oral swabs for antigen-targeting assays. Antibody-focused approaches could target IgM and IgG serology and/or IgA by mucosal or group oral fluid sampling. The utility of group-level oral fluids as a diagnostic sample has been expanding and the noninvasive approach that it allows is readily supported by the pig production industry [34]. Sampling by this manner is also of interest for zoonotic pathogens, such as BDBV, as it mitigates some biosafety concerns by eliminating the need for sharps and direct handling of the animals [35]. Further validation will be required to deploy any of these as diagnostic approaches for the detection of BDBV, including the development of reagents for use as controls; and future work is warranted to define the infection characteristics of BDBV infection in pigs, such as the effect of inoculum dose on the development of clinical disease, the duration and durability of the antibody response, the potential for pig-to-pig transmission, and the susceptibility of older sexually mature pigs. Although BDBV was not detected in muscle tissue during this study, the potential for BDBV contamination of other pork byproducts will also need to be evaluated to better inform further risk assessments.

Significant knowledge gaps remain when considering ebolavirus infection in swine. Future steps to better understand ebolavirus disease should include two avenues of research. First, experimental infection of additional ebolaviruses, particularly SUDV due to its high case fatality rate in humans and its importance in the context of public health should be evaluated. Second, when considering both past and current ebolavirus infection studies in swine, characterization of the host immune response in pigs should be pursued. In particular, the innate immune response in swine will provide insights into early host responses while also providing guidance to evaluate viral transmission and the zoonotic potential of these animals.

The role of pigs as a possible interim or amplifying host for ebolaviruses is a concern for both human public health and food security [13, 27]. Based on the available data from both natural and experimental infections, transmission to humans by direct contact with pigs or, theoretically, through contamination of pork products entering the food chain is possible [10, 12, 27]; therefore, a high index of suspicion should be maintained concerning the potential of a pig–ebolavirus relationship during future outbreaks [13, 18]. Importantly, in this study, pigs exhibited a subclinical infection with only two animals presenting a short-lived fever. This finding suggests that clinical suspicion alone may not be sufficient to detect infected pigs; and it supports the need for surveillance efforts to better understand the pathogen and host ecology while preparing for potential future zoonotic events, which could present undetected in regions with increased risk for virus spillover. Altogether, the results reported here support the addition of a third ebolavirus to the list of those capable of infecting domestic pigs, providing further corroboration to the hypothesis that pigs may play a role in the ecology of these high-consequence pathogens.

## Figures and Tables

**Table 1:** Detection of BDBV by real-time reverse transcription PCR of samples from experimentally infected pigs.

	Day post-inoculation												
Sample	0	1	2	3	4	5	6	7	8	9	10	11–21	22–28
Nasal swab	0/11	**9/11**	**9/11**	**9/11**	**10/11**	**7/9**	**6/9**	**2/7**	0/7	0/5	0/5	0/3	0/1
Oral swab	0/11	**6/11**	**2/11**	**3/11**	**3/11**	**3/7**	**1/9**	**1/7**	0/7	0/5	0/5	0/3	0/1
Rectal swab	0/11	**3/11**	**3/11**	0/11	0/11	0/7	0/9	0/7	0/7	0/5	0/5	0/3	0/1
Nasal wash	0/11	**10/11**	**8/11**	**8/11**	**8/11**	**6/7**	**6/9**	**5/7**	**4/7**	**2/5**	**1/5**	0/3	0/1
Blood	0/11	0/11	0/11	0/11	0/11	0/9	0/9	0/7	0/7	0/5	0/5	0/3	0/1

Results reported as number positive/number of samples collected at that time point. The sham-inoculated transmission control animal is excluded from this table as all results were negative. Bold text indicates clinically significant findings. Underlined text indicates that infectious virus was recovered from at least one sample at that time point. BDBV, Bundibugyo virus.

**Table 2:** Detection of BDBV by real-time reverse transcription PCR of group oral fluid samples from experimentally infected pigs.

dpi	No. samples tested	No. samples positive
0	2	0
1	2	**2**
2	2	**1**
3	2	**2**
4	2	**2**
5	2	**1**
6	2	**2**
7	2	**2**
8	2	**1**
9	1	0
10	1	0
11	1	0
12	1	0
13	1	0
14	1	0
21	1	0
28	1	0

Oral fluid samples were collected from group shared cotton ropes placed in pens. Bold text indicates clinically significant results. Underlined text indicates successful recovery of infectious virus by cell culture isolation from at least one sample at the indicated time point. dpi, days post-inoculation; BDBV, Bundibugyo virus.

**Table 3:** Virus-specific IgM antibody development in serum from pigs experimentally infected with BDBV.

	Days post-inoculation																
Pig ID	0	1	2	3	4	5	6	7	8	9	10	11	12	13	14	21	28
19-15	—	—	—	—	—	—	—	—	—	—	—	—	—	—	—	—	—
19-16	—	—	—	—	—	—	—	—	—	—	—	—	—	—	—	—	—
19-17	—	—	—	—	—	—	—	—	—	—	—	—	—	—	—	—	—
19-18	—	—	—	—	—	—	—	—	—	—	—	—	—	—	—	—	—
19-19	—	—	—	—	—	—	**100**	**400**	**1600**	—	—	—	—	—	—	—	—
19-20	—	—	—	—	—	—	—	**100**	**400**	—	—	—	—	—	—	—	—
20-20	—	—	—	—	—	—	—	—	—	**100**	**100**	—	—	—	—	—	—
20-21	—	—	—	—	—	—	—	—	**100**	**100**	**400**	—	—	—	—	—	—
20-22	—	—	—	—	—	—	—	—	—	—	—	**100**	**100**	**100**	**100**	—	—
20-23	—	—	—	—	—	—	—	**100**	**100**	**400**	**400**	**400**	**400**	**400**	**100**	**100**	—
20-24	—	—	—	—	—	—	—	—	—	—	—	**100**	**100**	**100**	**100**	**100**	—
20-25	—	—	—	—	—	—	—	—	—	—	—	—	—	—	—	—	—

Results reported as 1:titer determined for that animal at that time point. Bold text indicates clinically significant result. IgM, immunoglobulin M; BDBV, Bundibugyo virus.

**Table 4:** Virus-specific IgG antibody development in serum from pigs experimentally infected with BDBV.

	Days post-inoculation																
Pig ID	0	1	2	3	4	5	6	7	8	9	10	11	12	13	14	21	28
19-15	—	—	—	—	—	—	—	—	—	—	—	—	—	—	—	—	—
19-16	—	—	—	—	—	—	—	—	—	—	—	—	—	—	—	—	—
19-17	—	—	—	—	—	—	—	—	—	—	—	—	—	—	—	—	—
19-18	—	—	—	—	—	—	—	—	—	—	—	—	—	—	—	—	—
19-19	—	—	—	—	—	—	**100**	**400**	**400**	—	—	—	—	—	—	—	—
19-20	—	—	—	—	—	—	**100**	**400**	**400**	—	—	—	—	—	—	—	—
20-20	—	—	—	—	—	—	—	—	—	**100**	**400**	—	—	—	—	—	—
20-21	—	—	—	—	—	—	—	**100**	**100**	**400**	**400**	—	—	—	—	—	—
20-22	—	—	—	—	—	—	—	—	—	**100**	**100**	**400**	**400**	**400**	**400**	**100**	—
20-23	—	—	—	—	—	—	—	—	**100**	**400**	**400**	**800**	**800**	**1600**	**1600**	**800**	—
20-24	—	—	—	—	—	—	—	—	—	—	**100**	**100**	**400**	**400**	**400**	**100**	**100**
20-25	—	—	—	—	—	—	—	—	—	—	—	—	**100**	**100**	**100**	**100**	—

Results reported as 1:titer determined for that animal at that time point. Bold text indicates clinically significant result. IgG, immunoglobulin G; BDBV, Bundibugyo virus.

**Table 5:** Virus-specific IgA antibody development in nasal wash fluid from pigs experimentally infected with BDBV.

	Days post-inoculation																
Pig ID	0	1	2	3	4	5	6	7	8	9	10	11	12	13	14	21	28
19-15	—	—	—	—	—	—	—	—	—	—	—	—	—	—	—	—	—
19-16	—	—	—	—	—	—	—	—	—	—	—	—	—	—	—	—	—
19-17	—	—	—	—	—	—	—	—	—	—	—	—	—	—	—	—	—
19-18	—	—	—	—	—	—	—	—	—	—	—	—	—	—	—	—	—
19-19	—	—	—	—	—	—	**4**	**4**	**8**	—	—	—	—	—	—	—	—
19-20	—	—	—	—	—	—	—	**4**	**8**	—	—	—	—	—	—	—	—
20-20	—	—	—	—	—	—	—	—	**4**	**4**	**4**	—	—	—	—	—	—
20-21	—	—	—	—	—	—	—	—	—	—	**4**	—	—	—	—	—	—
20-22	—	—	—	—	—	—	—	—	—	—	—	**4**	**4**	**8**	**8**	**4**	—
20-23	—	—	—	—	—	—	—	—	—	4	8	**16**	**16**	**8**	**16**	**16**	—
20-24	—	—	—	—	—	—	—	—	—	—	—	—	—	—	—	—	—
20-25	—	—	—	—	—	—	—	—	—	—	—	—	—	**4**	—	—	—

Results reported as 1:titer determined for that animal at that time point. Bold text indicates clinically significant result. IgA, immunoglobulin A; BDBV, Bundibugyo virus.

**Table 6:** Virus-specific IgA antibody development in group oral fluids from pigs experimentally infected with BDBV.

dpi	Cohort 1	Cohort 2
0–7	—	—
8	**4**	**—**
9	—	—
10	—	**4**
11	—	**4**
12	—	**4**
13	—	**8**
14	—	**8**
15	—	**8**
16	—	**8**
17	—	**4**
18	—	**8**
19	—	**4**
20	—	—
21	—	**4**
22–28	—	—

Oral fluid samples were collected from group shared cotton ropes placed in pens. Results reported as 1:titer determined for that sample at that time point. Bold text indicates clinically significant results. IgA, immunoglobulin A; dpi, days post-inoculation; BDBV, Bundibugyo virus.

## Data Availability

The data used to support the findings of this study are included within the article.

## Supplementary Materials

Supplementary 1 Table S1: additional data outlining animal temperatures, pulse, and oxygen saturation. Table S2: clinical pathology findings in BDBV-infected pigs. Table S3: RT-PCR data for viral shedding. Table S4: additional virus sequence data.

Supplementary 2 Figure S1: additional lung histopathology. Figure S2: additional antibody titer data.

## References

[1] Kuhn J. H., Amarasinghe G. K., Basler C. F. (2019). ICTV virus taxonomy profile: filoviridae. *Journal of General Virology*.

[2] Kuhn J. H., Amarasinghe G. K., Perry D. L. (2020). *Filoviridae*.

[3] Goldstein T., Anthony S. J., Gbakima A. (2018). The discovery of Bombali virus adds further support for bats as hosts of ebolaviruses. *Nature Microbiology*.

[4] Towner J. S., Sealy T. K., Khristova M. L. (2008). Newly discovered ebola virus associated with hemorrhagic fever outbreak in Uganda. *PLoS Pathogens*.

[5] Wamala J. F., Lukwago L., Malimbo M. (2010). Ebola hemorrhagic fever associated with novel virus strain, Uganda, 2007–2008. *Emerging Infectious Diseases*.

[6] Kratz T., Roddy P., Oloma A. T. (2015). Ebola virus disease outbreak in Isiro, Democratic Republic of the Congo, 2012: signs and symptoms, management and outcomes. *PLOS ONE*.

[7] Leendertz S. A. J., Gogarten J. F., Düx A., Calvignac-Spencer S., Leendertz F. H. (2016). Assessing the evidence supporting fruit bats as the primary reservoirs for Ebola viruses. *EcoHealth*.

[8] Atherstone C., Diederich S., Pickering B. (2021). Investigation of Ebolavirus exposure in pigs presented for slaughter in Uganda. *Transboundary and Emerging Diseases*.

[9] Barrette R. W., Metwally S. A., Rowland J. M. (2009). Discovery of swine as a host for the *Reston ebolavirus*. *Science*.

[10] Miranda M. E. G., Miranda N. L. J. (2011). Reston ebolavirus in humans and animals in the Philippines: a review. *The Journal of Infectious Diseases*.

[11] Kobinger G. P., Leung A., Neufeld J. (2011). Replication, pathogenicity, shedding, and transmission of Zaire ebolavirus in pigs. *The Journal of Infectious Diseases*.

[12] Weingartl H. M., Embury-Hyatt C., Nfon C., Leung A., Smith G., Kobinger G. (2012). Transmission of Ebola virus from pigs to non-human primates. *Scientific Reports*.

[13] WHO (2009). Experts consultation on Ebola Reston pathogenicity in humans.

[14] Pan Y., Zhang W., Cui L., Hua X., Wang M., Zeng Q. (2014). Reston virus in domestic pigs in China. *Archives of Virology*.

[15] Fischer K., Jabaty J., Suluku R. (2018). Serological evidence for the circulation of Ebolaviruses in pigs from Sierra Leone. *The Journal of Infectious Diseases*.

[16] Fischer K., Camara A., Troupin C. (2020). Serological evidence of exposure to ebolaviruses in domestic pigs from Guinea. *Transboundary and Emerging Diseases*.

[17] Atherstone C., Galiwango R. G., Grace D. (2019). Analysis of pig trading networks and practices in Uganda. *Tropical Animal Health and Production*.

[18] Atherstone C., Smith E., Ochungo P., Roesel K., Grace D. (2017). Assessing the potential role of pigs in the epidemiology of Ebola virus in Uganda. *Transboundary and Emerging Diseases*.

[19] FAO (2011). *Mapping Supply and Demand for Animal-Source Foods to 2030*.

[20] Pickering B. S., Collignon B., Smith G., Marszal P., Kobinger G., Weingartl H. M. (2018). Detection of Zaire ebolavirus in swine: assay development and optimization. *Transboundary and Emerging Diseases*.

[21] Pickering B. S., Smith G., Pinette M. M. (2021). Susceptibility of domestic swine to experimental infection with severe acute respiratory syndrome coronavirus 2. *Emerging Infectious Diseases*.

[22] Katoh K., Standley D. M. (2013). MAFFT multiple sequence alignment software version 7: improvements in performance and usability. *Molecular Biology and Evolution*.

[23] Kearse M., Moir R., Wilson A. (2012). Geneious basic: an integrated and extendable desktop software platform for the organization and analysis of sequence data. *Bioinformatics*.

[24] Huang Y., Niu B., Gao Y., Fu L., Li W. (2010). CD-HIT Suite: a web server for clustering and comparing biological sequences. *Bioinformatics*.

[25] Quick J., Grubaugh N. D., Pullan S. T. (2017). Multiplex PCR method for MinION and illumina sequencing of Zika and other virus genomes directly from clinical samples. *Nature Protocols*.

[26] Lewis C. E., Pickering B. (2020). Livestock and risk group 4 pathogens: researching zoonotic threats to public health and agriculture in maximum containment. *ILAR Journal*.

[27] Haddock E., Saturday G., Feldmann F. (2021). Reston virus causes severe respiratory disease in young domestic pigs. *Proceedings of the National Academy of Sciences of the United States of America*.

[28] Marsh G. A., Haining J., Robinson R. (2011). Ebola Reston virus infection of pigs: clinical significance and transmission potential. *The Journal of Infectious Diseases*.

[29] Nfon C. K., Leung A., Smith G., Embury-Hyatt C., Kobinger G., Weingartl H. M. (2013). Immunopathogenesis of severe acute respiratory disease in *Zaire ebolavirus*-infected pigs. *PLoS ONE*.

[30] Ebihara H., Takada A., Kobasa D. (2006). Molecular determinants of Ebola virus virulence in mice. *PLoS Pathogens*.

[31] Leroy E. M., Baize S., Mavoungou E., Apetrei C. (2002). Sequence analysis of the GP, NP, VP40 and VP24 genes of Ebola virus isolated from deceased, surviving and asymptomatically infected individuals during the 1996 outbreak in Gabon: comparative studies and phylogenetic characterization. *Journal of General Virology*.

[32] Leroy E. M., Baize S., Volchkov V. E. (2000). Human asymptomatic Ebola infection and strong inflammatory response. *The Lancet*.

[33] Rasmussen A. L., Okumura A., Ferris M. T. (2014). Host genetic diversity enables Ebola hemorrhagic fever pathogenesis and resistance. *Science*.

[34] Bjustrom-Kraft J., Christopher-Hennings J., Daly R. (2018). The use of oral fluid diagnostics in swine medicine. *Journal of Swine Health and Production*.

[35] Formenty P., Leroy E. M., Epelboin A. (2006). Detection of Ebola virus in oral fluid specimens during outbreaks of Ebola virus hemorrhagic fever in the Republic of Congo. *Clinical Infectious Diseases*.

[36] Lewis C. E. (2022). *Susceptibility of domestic pigs to experimental infection with ebolaviruses*.

