# Correction to “Experimental Infection of Bundibugyo Virus in Domestic Swine Leads to Viral Shedding with Evidence of Intraspecies Transmission”

**DOI:** 10.1155/tbed/9826576

**Published:** 2026-07-03

C. E. Lewis, M. M Pinette, S. M. Lakin, et al., “Experimental Infection of Bundibugyo Virus in Domestic Swine Leads to Viral Shedding with Evidence of Intraspecies Transmission,” *Transboundary and Emerging Diseases*, 2024, 5350769, https://doi.org/10.1155/2024/5350769.

In the article, there is an error in the 0.4 µM BDBV L‐gene forward primer sequence reported in Section 2.11. The correct sequence is below:

BDBV L‐gene Forward 1: 5′‐TCGGGAAGATCCCCCGGAAG‐3′.

We apologize for this error.

