# Supplementary material for: Experimental Infection of Bundibugyo Virus in Domestic Swine Leads to Viral Shedding with Evidence of Intraspecies Transmission
Source: Transbound Emerg Dis. 2024 Jan 9;2024:5350769. doi: 10.1155/2024/5350769 (PMC12017203; doi:10.1155/2024/5350769)
Supplement: Supplementary 2 — Figure S1: additional lung histopathology. Figure S2: additional antibody titer data. [file 5350769.f2.docx]

**10 dpi 21 dpi 28 dpi**


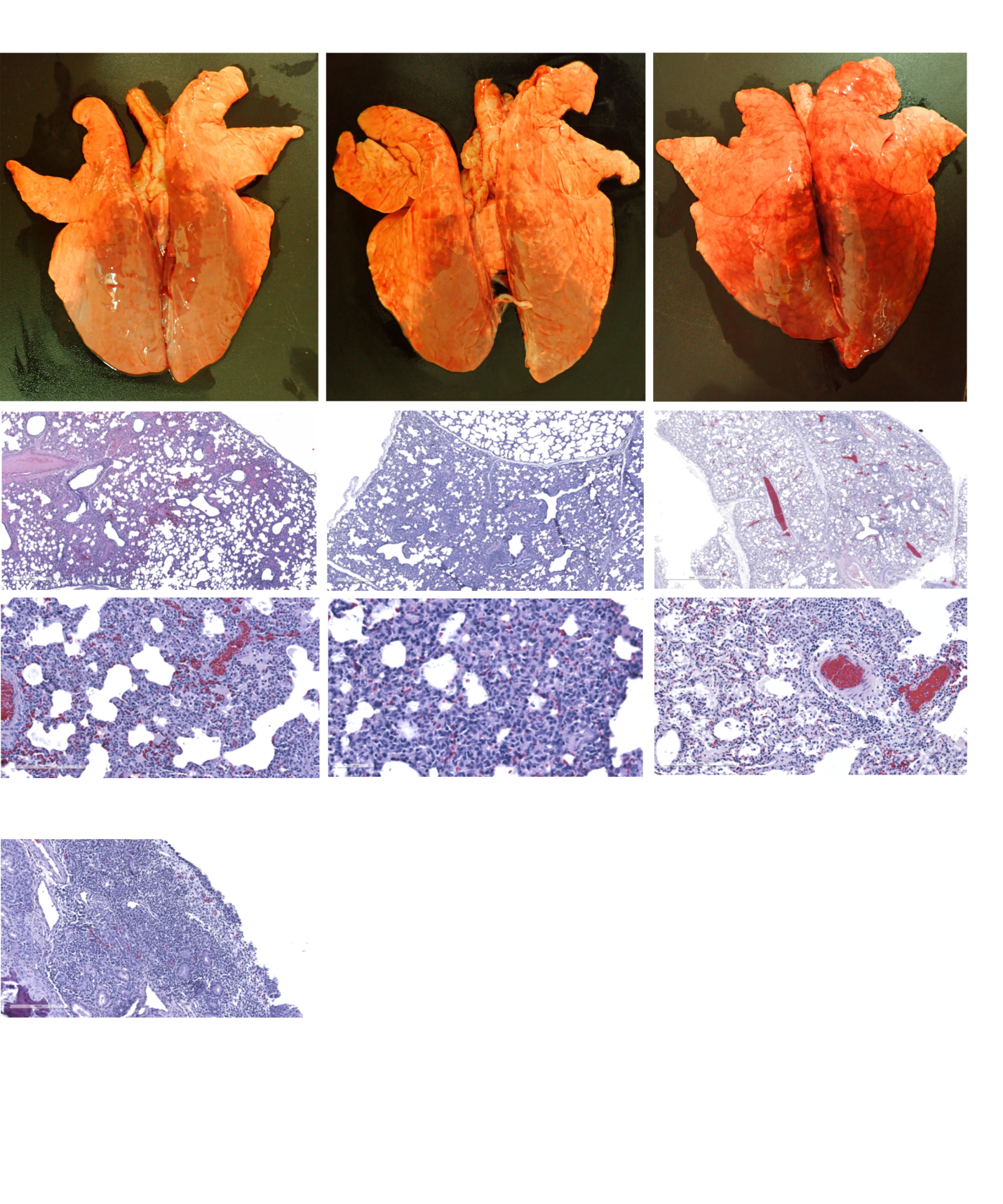


i.

h.

e.

f.

c.

b.

g.

d.

a.

**Supplement Figure 1. Lung pathology in BDBV-infected pigs at ten, twenty-one, and twenty-eight dpi.** Beginning at ten dpi, areas of reddening of the dorsal aspect of the caudal lung lobes was present during gross examination at necropsy (a, d, g). These areas were not consistent with gross lesions at earlier time points and we suspect that this represents artifactual congestion secondary to euthanasia. At ten dpi, mild to moderate interstitial infiltration with alveolar septal expansion involving inflammatory cells was noted during histopathologic evaluation (b, c), but the associated pneumonia was less severe compared to earlier time points. Multifocal areas of mild interstitial infiltration were still present at twenty-one (e, f) and twenty-eight (h, i) dpi. BDBV, Bundibugyo virus; dpi, day post-inoculation.


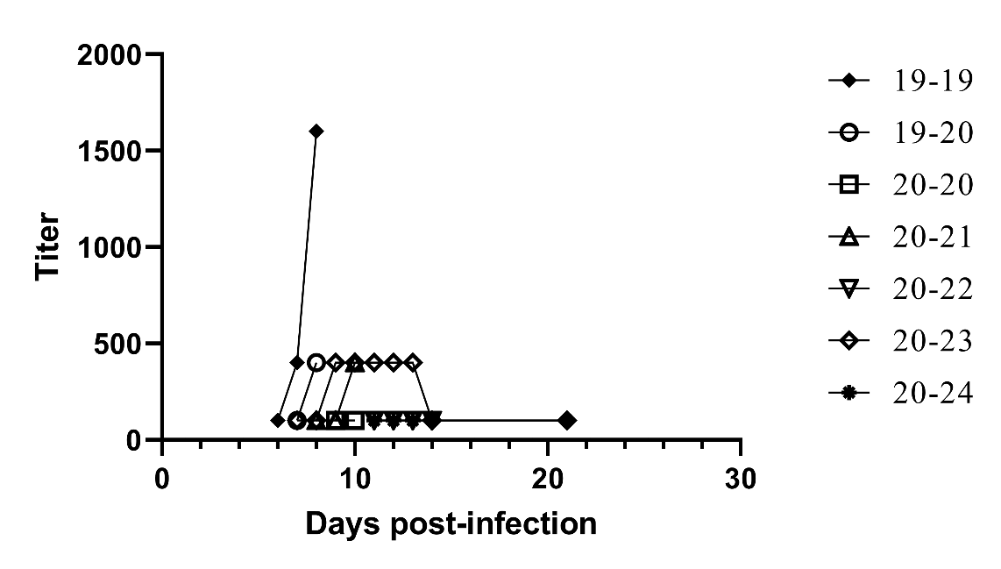

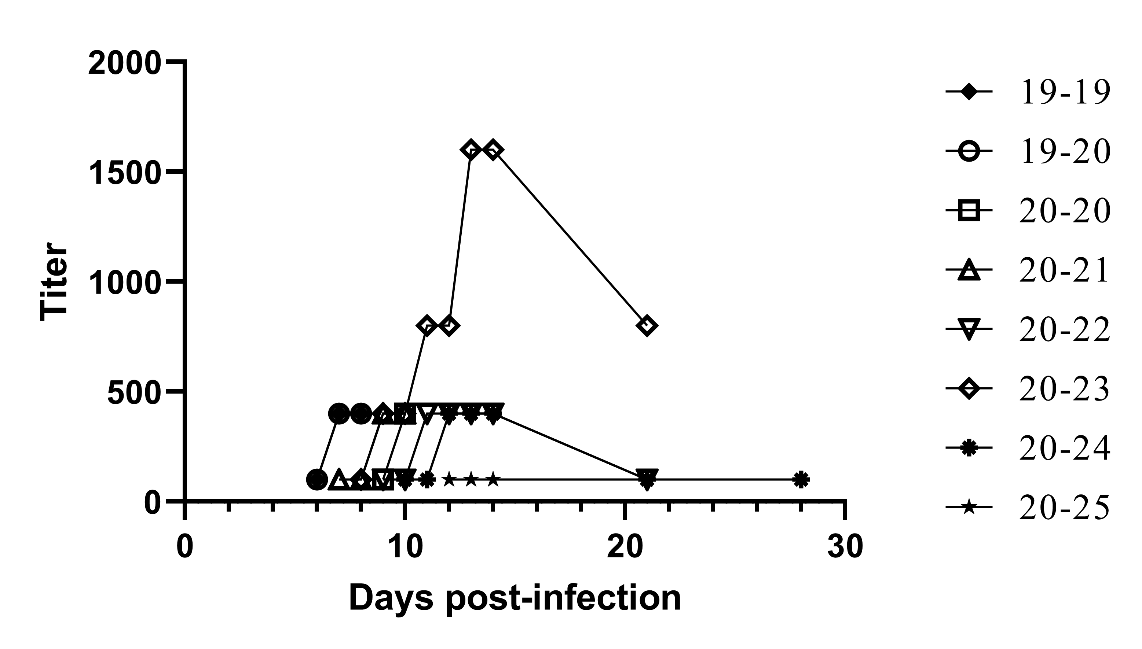


**a**

**b**

**Supplement Figure 2. Virus-specific IgM and IgG development in serum.**

Antibody titers for pigs experimentally infected with BDBV;panel a) IgM antibody titers, panel b) IgG antibody titers.
